# Nicotine deprivation elevates neural representation of smoking-related cues in object-sensitive visual cortex: a proof of concept study

**DOI:** 10.1007/s00213-017-4628-3

**Published:** 2017-04-21

**Authors:** Anne Havermans, Onno C. P. van Schayck, Eric F. P. M. Vuurman, Wim J. Riedel, Job van den Hurk

**Affiliations:** 1 Department of Neuropsychology and Psychopharmacology, Faculty of Psychology and Neuroscience, Maastricht University, Maastricht, the Netherlands; 2 CAPHRI School for Public Health and Primary Care, Maastricht, the Netherlands; 3 Department of Cognitive Neuroscience, Faculty of Psychology and Neuroscience, Maastricht University, Maastricht, the Netherlands; 4 Maastricht Brain Imaging Center (MBIC), Maastricht, the Netherlands; 5 Laboratory of Biological Psychology, University of Louvain, Louvain, Belgium

**Keywords:** Attention bias, Lateral occipital complex, Nicotine deprivation, Pattern classification, Smoking cues

## Abstract

**Objective:**

In the current study, we use functional magnetic resonance imaging (fMRI) and multi-voxel pattern analysis (MVPA) to investigate whether tobacco addiction biases basic visual processing in favour of smoking-related images. We hypothesize that the neural representation of smoking-related stimuli in the lateral occipital complex (LOC) is elevated after a period of nicotine deprivation compared to a satiated state, but that this is not the case for object categories unrelated to smoking.

**Methods:**

Current smokers (≥10 cigarettes a day) underwent two fMRI scanning sessions: one after 10 h of nicotine abstinence and the other one after smoking ad libitum. Regional blood oxygenated level-dependent (BOLD) response was measured while participants were presented with 24 blocks of 8 colour-matched pictures of cigarettes, pencils or chairs. The functional data of 10 participants were analysed through a pattern classification approach.

**Results:**

In bilateral LOC clusters, the classifier was able to discriminate between patterns of activity elicited by visually similar smoking-related (cigarettes) and neutral objects (pencils) above empirically estimated chance levels only during deprivation (mean = 61.0%, chance (permutations) = 50.0%, *p* = .01) but not during satiation (mean = 53.5%, chance (permutations) = 49.9%, ns.). For all other stimulus contrasts, there was no difference in discriminability between the deprived and satiated conditions.

**Conclusion:**

The discriminability between smoking and non-smoking visual objects was elevated in object-selective brain region LOC after a period of nicotine abstinence. This indicates that attention bias likely affects basic visual object processing.

## Introduction

While tobacco addiction is often regarded as the direct result of the pharmacological effects of nicotine, there are various other processes involved as well. For instance, a critical role in the maintenance of tobacco addiction is reserved for the interaction between environment and corresponding neural events; i.e. drug-related cues in the environment are paired with the rewarding physiological effects of nicotine. After repeated pairing and reinforcement of these cues, they become highly salient, triggering the urge to smoke (Robinson and Berridge 2008; Benowitz 2010). In this process, these motivationally relevant smoking cues have been shown to automatically and involuntarily capture the smoker’s selective attention (Mogg and Bradley 2002; Hogarth et al. 2003; Field and Cox 2008). The resulting attention bias exhibited by smokers is associated with increased craving and has been implicated in maintaining addictive behaviour and provoking relapses (Bradley et al. 2003; Janes et al. 2010a; Austin and Duka 2012). Additionally, nicotine abstinence has been shown to increase subjective craving in response to smoking cues and enhance attention bias, making it even more difficult for (ex-)smokers to remain abstinent (Gross et al. 1993; Sayette and Hufford 1994; Waters and Feyerabend 2000; Zack et al. 2001; Field et al. 2004). Thus, even existing smoking cessation treatments will be less effective as long as smokers are still automatically being attracted to these craving-eliciting cues. Accordingly, there is a need for new (additional) treatments targeting extinction of automatic responses to smoking cues. More knowledge about the exact underlying mechanisms can be essential in the process of developing those.

Functional imaging studies have shown that craving-provoking drug cues elicit a response in a network of frontal brain regions, mainly consisting of the amygdala, anterior cingulate cortex (ACC), dorsolateral prefrontal cortex (dlPFC) and orbitofrontal cortex (OFC) (Wilson et al. 2004). This is not surprising, since these areas are connected to the dopaminergic mesolimbic reward pathway which becomes activated in response to actual drug exposure (Brody 2006). The amygdala is believed to enhance identification of emotionally salient stimuli (like conditioned drug cues) (Phillips et al. 2003), whereas the areas in the prefrontal cortex play a key role in the guidance of goal-directed and motivational behaviour. Specifically, the OFC is thought to integrate and modulate activity from several limbic areas (such as the amygdala) involved in reward processing (Volkow and Fowler 2000), and the dlPFC is implicated in regulatory processing and decision-making (Watanabe et al. 2002). The ACC, in addition, has been associated with conflict monitoring in the presence of competing response alternatives (Kerns et al. 2004). Reactivity of these areas to smoking cues may therefore reflect a process of deciding whether or not to resist the urge to smoke. Although findings are somewhat inconsistent across studies, increased activation in response to smoking cues is mostly reported in brain regions related to reward and motivational processing as well as in the frontal and parietal attentional networks (David et al. 2005; Brody et al. 2007; Franklin et al. 2007; Rubinstein et al. 2010; Luijten et al. 2011; Claus et al. 2013). Activation of these regions in response to smoking cues is evident, since it reflects the rewarding value of the cues and the motivational and attentional processes guiding drug-seeking behaviour (Engelmann et al. 2012).

Nonetheless, these areas are located relatively late in the pathway through which visual information is processed. That is, impulses are first transmitted from the primary visual cortex via the extrastriate cortex to higher-order visual association areas. From there, projections finally go to multimodal processing regions including the amygdala and prefrontal cortex (Tanaka 1992). Interestingly, return connections of the amygdala to various levels of the ventral stream are thought to enhance sensory processing of emotionally salient stimuli in the visual cortex (Amaral et al. 2003). So although salience attribution is situated in higher-processing stages (Goldstein and Volkow 2011), it may well influence earlier visual processing in a top-down fashion. Even more specifically, a study by Murray and Wojciulik (2004) has shown that top-down attention increased not just activity but also neural selectivity in the lateral occipital complex, a region involved in the visual processing of objects and shapes (Murray and Wojciulik 2004). Thus, one mechanism by which a smoker’s attention could be biased towards (salient) visual smoking cues may be via increased processing in the extended visual system.

In accordance with this hypothesis, there is some evidence for increased activation in areas of the primary visual and extrastriate cortices in response to smoking cues (Due et al. 2002; Janes et al. 2010b; Engelmann et al. 2012; Havermans et al. 2014). Nevertheless, the evidence remains scarce, and existing theories on attention bias have explicitly argued for a role of the brain’s reward pathway and not the extended visual system in this process (Everitt and Robbins 2005; Robinson and Berridge 2008). A reason for the inconclusive neuroimaging evidence for the involvement of the visual system could be that visual processing of salient smoking cues is only slightly enhanced compared to neutral cues, since no motivational value has been attributed in this stage of processing. Standard univariate functional magnetic resonance imaging (fMRI) analysis techniques might not be sufficiently sensitive to detect such subtle effects. Furthermore, in several of these cue reactivity studies, the participants had been smoking before scanning (Janes et al. 2010b; Engelmann et al. 2012). Since smoking cues are of greater salience and relevance for deprived smokers than for satiated smokers (Robinson and Berridge 2008), this may have minimized the saliency—and thereby the enhanced visual processing—of the smoking cues.

In the current study, we use functional MRI and multi-voxel pattern analysis (MVPA) to investigate basic visual processing of smoking-related objects in tobacco-addicted participants. This method allows us to consider the pattern of activity of the total amount of voxels in the lateral occipital complex (LOC), instead of analysing each voxel independently and looking at the average response of the whole region. By employing the spatial distribution of neural responses in this way, pattern analysis techniques are able to pick up information that is too subtle to be discovered by traditional univariate analyses (De Martino et al. 2008; Mur et al. 2009; Mahmoudi et al. 2012; Tong and Pratte 2012; Woolgar et al. 2016). In the current study, we use functional MRI and a linear classification algorithm (support vector machine (SVM); Cortes and Vapnik 1995) to investigate whether smoking-related visual cues are processed differently when a smoker is deprived of nicotine compared to a satiated state. We investigated the discriminability of neural responses to cigarette images and visually comparable pencil images immediately after the participant has smoked a cigarette, and compare this to the discriminability of the responses when the smoker is craving for nicotine. We hypothesize that the neural responses will grow more distinct for the smoking-related cues in the latter situation, as these stimuli will be increasingly behaviourally relevant. This in turn would imply that nicotine deprivation in smokers directly biases early visual object recognition towards smoking-related cues.

## Methods

### Participants

Fourteen right-handed, current smokers (6 males, mean age 25.21 years) participated in this study. Individuals who reported smoking a minimum of 10 cigarettes a day on average for at least 1 year were included. Exclusion criteria were history of physical or mental illness, use of psychotropic medication, history of drug or alcohol abuse, and contraindications for the MRI scanner. Participants received financial compensation for their participation and travel costs. The study was approved by the Ethical Review Committee Psychology and Neuroscience of Maastricht University, and written informed consent was obtained from each participant.

### Procedure

Participants were scanned on two occasions. On one occasion, they were instructed to refrain from smoking for at least 10 h before the experiment. On the other occasion, participants were allowed to smoke ad libitum and were specifically asked to smoke a cigarette just before entering the lab in order to achieve maximal satiation. The order of the sessions was randomized across subjects. Upon entering the lab, smoking status was verified by exhaled carbon monoxide measurement. In addition, participants reported at what time they had smoked their most recent cigarette. Furthermore, they filled out the Fagerström Test for Nicotine Dependence (FTND) (Heatherton et al. 1991), the Minnesota Nicotine Withdrawal Scale (MNWS) (Cappelleri et al. 2005) and an MRI eligibility check.

### Localizer run

An independent functional localizer run preceded the experiment to localize object-sensitive regions of the brain. Participants were presented with 12 blocks of 10-Gy-scale pictures of common objects and 12 blocks of scrambled versions of the same pictures, all equalized for luminance. All pictures were presented for 1200 ms with an inter-stimulus interval of 600 ms. Stimulus blocks were presented in random order and interleaved with fixation blocks of 12,600 ms.

### Experimental run

**Fig. 1:**
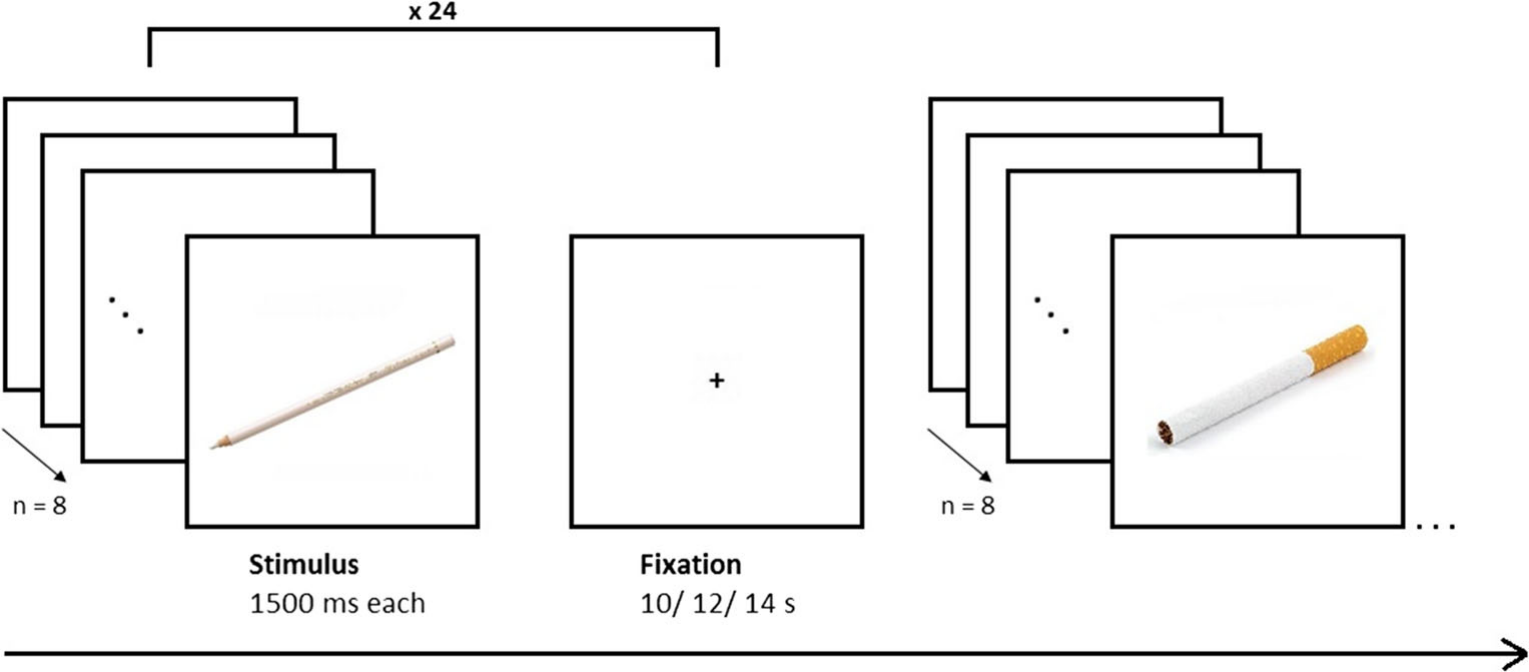
Experimental task paradigm*.* Participants were presented with eight stimulus blocks consisting of eight pictures of either cigarettes or pencils or chairs. The stimulus blocks were presented in random order and interchanged with intervals of 10, 12 or 14 s of rest

Stimuli for this run consisted of coloured pictures of cigarettes, pencils and chairs in similar colours, presented centrally on a white background. Participants were presented with three runs, consisting of eight blocks of eight pictures of either cigarettes or pencils or chairs. Pictures were presented for 1500 ms with a 50-ms inter-stimulus interval. Stimulus blocks were presented in random order and interchanged with intervals of 10, 12 or 14 s of rest (see Fig. 1). These intervals allowed the blood oxygenated level-dependent (BOLD) response elicited by the previous stimulus block to return back to baseline before the onset of the adjacent block. To ensure attention to the pictures, participants had to respond to catch trials in which presentation of the preceding picture was repeated.

### Imaging data acquisition

Functional and anatomical images were acquired in a 3-T Siemens Prisma Scanner (Siemens Medical Solutions, Germany) using a 64-channel head coil. For the localizer run, functional images were obtained for 33 contiguous axial slices (voxel size = 3 × 3 × 3 mm) using a standard gradient echo planar imaging (EPI) sequence (repetition time [TR] = 1800 ms, echo time [TE] = 30 ms, matrix size = 72 × 72, flip angle [FA] = 77°). Functional images for the experimental runs were also acquired with an EPI sequence (TR = 2000 ms, TE = 30 ms, matrix size = 72 × 72, FA = 77°), but for 36 contiguous axial slices (voxel size = 3 × 3 × 3 mm). Both functional measurements covered as much as possible the entire cortical volume. Whole brain structural images were acquired using a 1 × 1 × 1 mm resolution T1-weighted MPRAGE sequence ([TR] = 2250 ms, [TE] = 2.21 ms, [FA] = 9°). The first two volumes of all functional runs were discarded because of possible T1 saturation.

Participants were placed in supine position on the scanner bed, and their heads were fixated with foam pads. Stimuli were projected on a screen at the back of the scanner bore, and were visible to the participants through a mirror attached to the head coil. Responses were made with a hand-held button box. Stimulus presentation was accomplished with Presentation software (Neurobehavioral Systems Inc., Albany, CA) for the localizer run and E-Prime® (Psychology Software Tools, Inc. Sharpsburg, PA) for the experimental runs and was synchronized to the MR data acquisition.

### Analyses

Imaging data was pre-processed and analysed using BrainVoyager QX version 2.8 (Brain Innovation, Maastricht, The Netherlands) and Matlab R2014b (MathWorks Inc., Natick, MA). First, functional images were corrected for possible susceptibility-induced EPI distortions by using B0 fieldmaps and the BrainVoyager QX plugin anatabacus (Breman et al. 2009). The functional data were then corrected for slice scan-time differences and 3D head motion (six parameters). In order to enhance the subsequent alignment of the functional images to the anatomical volume, the first and third runs were corrected with the second run as intra-session reference, as the acquisition of this run was temporally adjacent to the anatomical scan. Subsequently, linear trends and low-frequency temporal drifts were removed from the data using a high-pass filter, removing temporal frequencies below 6 cycles per run. The resulting functional data were co-registered to the anatomical volume and transformed to Talairach space. To further reduce the effects of motion-related variability (‘spikes’), a custom Matlab algorithm was applied that for each volume computed the percentage of voxels exceeded the voxel mean intensity by more than 4 standard deviations. Volumes with more than 2% extreme voxels were labelled as affected by ‘excessive’ motion. These volumes were replaced by new values derived from a voxel-wise spline interpolation between the volumes that temporally bordered the removed volumes. In case the excessive volumes were at the beginning or end of the run, the volumes were simply removed.

A standard whole brain univariate random effects GLM analysis was performed to localize bilateral LOC clusters for each subject. The locations of the object selective regions of interest (ROIs) were determined based on a conjunction contrast and anatomical criteria. We selected voxels within the bilateral ventral occipito-temporal cortex that showed a significant response (Bonferroni corrected) in the conjunction analysis between an object-responsive contrast (objects > scrambled objects) and an object-positive contrast (objects > baseline).

The experimental runs were analysed through a pattern classification approach (Cox and Savoy 2003) using custom written code in Matlab. The pseudocode of this analysis is provided as a supplement. This analysis was confined to the resulting voxels from the analysis of the localizer run. First, for each voxel within the region of interest, individual responses to the experimental blocks were estimated by fitting a double-gamma haemodynamic response function (HRF) to the voxel’s time course, using the resulting beta as block estimate. For each ROI and each condition, this resulted in a matrix with dimensions V × B, where V represents the number of voxels, and B stands for the number of blocks. These matrices capture the fine-grained neural information about the conditions that may be apparent in the response patterns. The blocks were labelled according to their corresponding condition (cigarette, pencil, chair) and normalized (*z*-scored) so as to obtain a mean response of 0 and standard deviation of 1 across all voxels within our region of interest.

The analysis was run using a leave-1-run-out cross-validation. This entails that the classification algorithm was trained on two out of three runs, and its generalization performance tested on the run that was left out. This procedure is repeated three times, ensuring that each run is once used for testing, after which the three prediction accuracies are averaged. First, for the cigarette and pencil conditions, the dataset was split into a training set consisting of two runs (i.e. 16 examples) per condition and a test set consisting of one run (i.e. 8 examples) per condition. The labelled training trials were submitted to a linear support vector machine classifier (C = 1) (Cortes and Vapnik 1995; Mourão-Miranda et al. 2005; Formisano et al. 2008), which performs binary classification on a dataset by placing all cases in a multidimensional space. Each individual block (or example) is expressed as a vector of N features (voxels) in the N-dimensional space. This creates two ‘clouds’ of block vectors in the multidimensional space, one for each condition tested. The algorithm then attempts to define an optimal separation boundary, or hyperplane, between the two classes, given the training data. The generalizability of the trained classifier is then subsequently assessed by feeding the independent and unlabelled test blocks from the left-out run to the algorithm. The accuracy at which the classifier is able to determine the correct labels from these trials given only the response patterns is an indication of successful learning of the algorithm. This, in turn, reflects a meaningful difference in spatial patterns of neural activity elicited by the two conditions.

In order to statistically quantify the prediction results, we empirically estimated the distribution of prediction accuracies under the null hypothesis by a bootstrapping approach: instead of training the classifier on voxel patterns and corresponding class labels, we pseudo randomly assigned the labels to the patterns (randomization of labels per run). The classifier then learned the arbitrary relationship between patterns and classes. By feeding the test trials to the model, we obtained a prediction accuracy under the null hypothesis. Repeating this 1000 times yields the distribution of prediction accuracies under the null hypothesis. This entire analysis was repeated for the cigarette and chair, and pencil and chair classes. Finally, a non-parametric Wilcoxon signed-rank test (Wilcoxon 1945) was performed to statistically test the mean prediction accuracies against the permutations, and to test the difference between the deprivation and satiation sessions. Resulting *p* values were corrected for multiple comparisons by computing the false discovery rate (FDR) using *q* = 0.05.

In order to test the variables for possible interaction effects, we computed the two-way *F*-statistic using a repeated-measures ANOVA with the factors deprivation state (2) × stimulus type (3). Then, we permuted the 2 × 3 condition labels across participants and recomputed the *F*-statistic in 10,000 iterations, creating the distribution of *F* under the null hypothesis (Suckling and Bullmore 2004). The probability of finding the true interaction *F*-statistic under the assumption that the H0 is true was subsequently derived from the permuted *F*-distribution. A priori planned comparisons between the satiated and deprived conditions were made for each stimulus category, regardless of the outcome of the corresponding overall interaction test. This is a legitimate procedure if the comparisons are suggested by the theoretical basis of the experiment (Winer 1971). Access to the Matlab codes used in this study will be provided upon individual request.

## Results

### Participant characteristics

Data of four participants had to be discarded because they did not fully complete all experimental runs. All data reported regard the final sample of 10 participants. The mean Fagerström score of our participants was 2.8 (SD = 1.79), which reflects a low (to moderate) level of smoking dependence (Heatherton et al. 1991). On average, participants smoked approximately 10 cigarettes a day, and there was no difference in the number of cigarettes smoked in the weeks preceding each session (mean deprived = 72.7 SD = 36.73; mean satiated = 76.1, SD = 35.22; *t*_(18)_ = −.211, *p* = .835). Time since last cigarette ranged from 5 to 50 min (mean = 17.5 min, SD = 14.77 min) for the satiated session and from 10 to 19 h (mean = 12.64 h, SD = 2.89 h) for the deprived session. Exhaled carbon monoxide values were significantly lower when participants had been deprived of smoking (range deprived 0–9, mean = 4.18; range satiated 5–13, mean = 8.00; *t*_(18)_ = −3.425, *p* = .004). Scores on the MNWS did not differ between the two sessions (mean deprived = 10.90, SD = 5.80; mean satiated = 10.40, SD = 6.92; *t*_(18)_ = .175, ns.). Moreover, participants did not report more craving after smoking deprivation (as indicated on a 0–4 scale; mean deprived = 2.20, SD = 0.92; mean satiated = 1.70, SD = 1.25; *t*_(18)_ = 1.018, ns.).

### Localizer

**Fig. 2:**
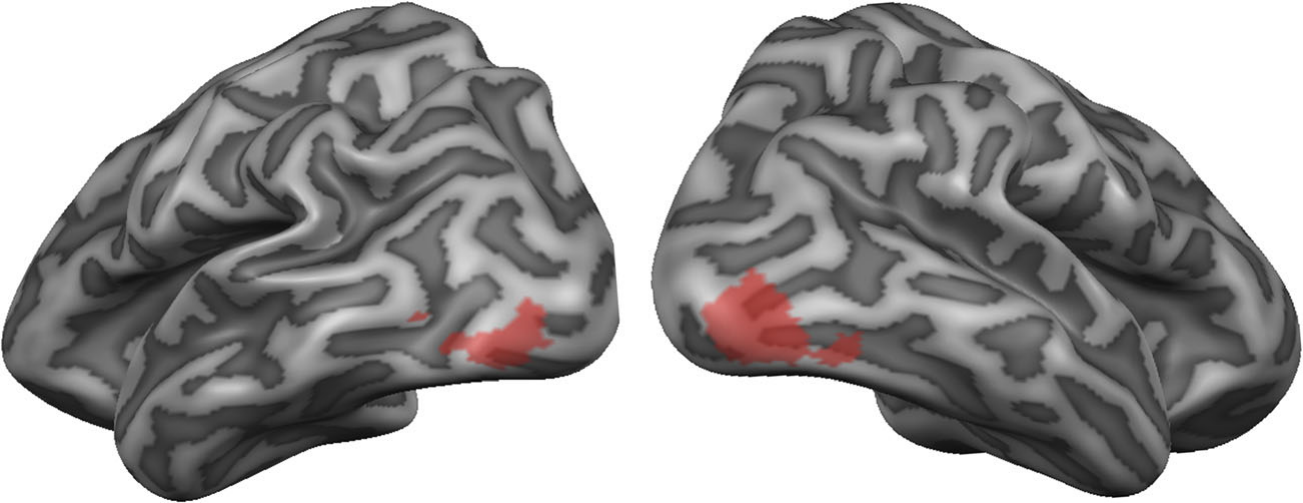
Average LOC clusters. Clusters of object-sensitive voxels within bilateral ventral occipito-temporal cortex, averaged over all participants. Projected on a partially inflated individual cortex reconstruction

Significant bilateral LOC clusters were identified in all but one participant. The average location of these clusters over all participants is shown in Fig. 2. For one participant, the specified contrast yielded no significant voxels with and without Bonferroni correction. Therefore, the average location of left and right LOC of all other participants was used in the MVPA analysis for this participant. The classification analysis was performed on both left and right LOC combined, as well as on separate left and right LOC voxels.

### Multi-voxel pattern analysis

#### Bilateral LOC

**Fig. 3:**
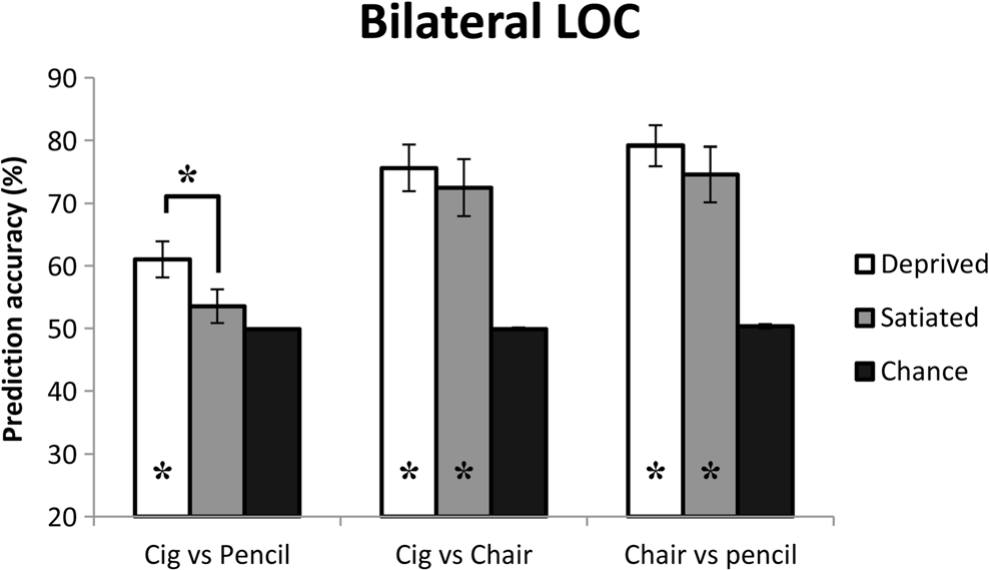
Classification results of all contrasts over bilateral LOC. Prediction accuracies for the cigarette vs pencil contrast were significantly higher when participants were deprived of smoking (*p* < .02). No differences between the deprived and satiated conditions were found for the other condition pairs. *Asterisks* indicate significant differences from permuted chance levels

Over the voxels of left and right LOC combined, the classification algorithm was able to discriminate between patterns of activity elicited by visually similar smoking-related (cigarettes) and neutral objects (pencils) above empirically estimated chance levels only when participants were smoking deprived (mean = 61.0%, mean permutation = 50.0%, *p* = .01 (uncorrected, *p* = .012 FDR-corrected)) but not when they were satiated (mean = 53.5%, mean permutation = 49.9%, ns.); see Fig. 3. Moreover, the difference in prediction accuracies between the satiated and deprived conditions was significantly larger than the difference between the permutation accuracies of both conditions (accuracies D-S = 7.50, permutations D-S = −0.12, *p* = .02 (uncorrected, *p* = .06 FDR-corrected)). Discrimination between the visually less similar smoking-related (cigarettes) and neutral (chairs) cues was possible above permutation chance levels when participants were deprived (mean = 75.6%, mean permutation = 50.0%, *p* = .002 (uncorrected, *p* = .012 FDR-corrected)) as well as when they were satiated (mean = 72.5%, mean permutation = 49.9%, *p* = .004 (uncorrected, *p* = .008 FDR-corrected)), and did not differ between conditions (accuracies D-S = 3.12, permutations D-S = 0.13, ns.). Similarly, discrimination between two visually distinct neutral objects (pencils, chairs) was possible above empirical chance levels during deprivation (mean = 79.2%, mean permutation = 50.0%, *p* = .002 (uncorrected, *p* = .006 FDR-corrected)) and satiety (mean = 74.6%, mean permutation = 50.3%, *p* = .004 (uncorrected, *p* = .006 FDR-corrected)) and also did not differ between the two conditions (accuracies D-S = 4.58, permutations D-S = −0.33, ns.). There was no significant interaction between the two deprivation states and the three stimulus combinations in bilateral LOC voxels (*p* > .1).

#### Left and right LOC

In left LOC, discrimination between response patterns elicited in response to visually similar smoking-related (cigarettes) and neutral objects (pencils) was possible above empirically estimated chance levels when participants were deprived of smoking (mean = 58.5%, mean permutation = 50%, *p* = .01 (uncorrected, *p* = .012 FDR-corrected)), but not when they were satiated (mean = 52.3%, mean permutation = 50%, ns.). However, the difference in decoding between the deprived and satiated conditions was significantly larger than the difference in empirically estimated chance levels (accuracies D-S = 6.25, permutations D-S = −.03, *p* = .01 (uncorrected, *p* = .06 FDR-corrected)). Moreover, the classifier was able to discriminate between responses elicited by the visually less similar smoking-related (cigarettes) and neutral (chairs) cues above permutation chance levels in the deprived (mean = 75%, mean permutation = 50%, *p* = .002 (uncorrected, *p* = .006 FDR-corrected)) as well as the satiated (mean = 73.3%, mean permutation = 50%, *p* = .002 (uncorrected, *p* = .006 FDR-corrected)) conditions. In addition, the classifier’s ability to discriminate between responses to cigarettes and chairs did not differ between deprivation and satiety (accuracies D-S = 1.67, permutations D-S = −.04, ns.). Finally, discrimination between two neutral objects based on responses in left LOC was possible when participants were satiated (mean 73.1%, mean permutation = 50.0%, *p* = .004 (uncorrected, *p* = .006 FDR-corrected)) and when they were deprived (mean = 75.8%, mean permutation = 50.0%, *p* = .002 (uncorrected, *p* = .006 FDR-corrected)). In addition, there was no significant difference in prediction accuracies for the neutral contrast (chairs–pencils), between the deprived and satiated states (accuracies D-S = 2.71, permutations D-S = 0.00, ns.) There was no significant interaction between the two deprivation states and the three stimulus combinations in left LOC (*p* > .1).

**Fig. 4:**
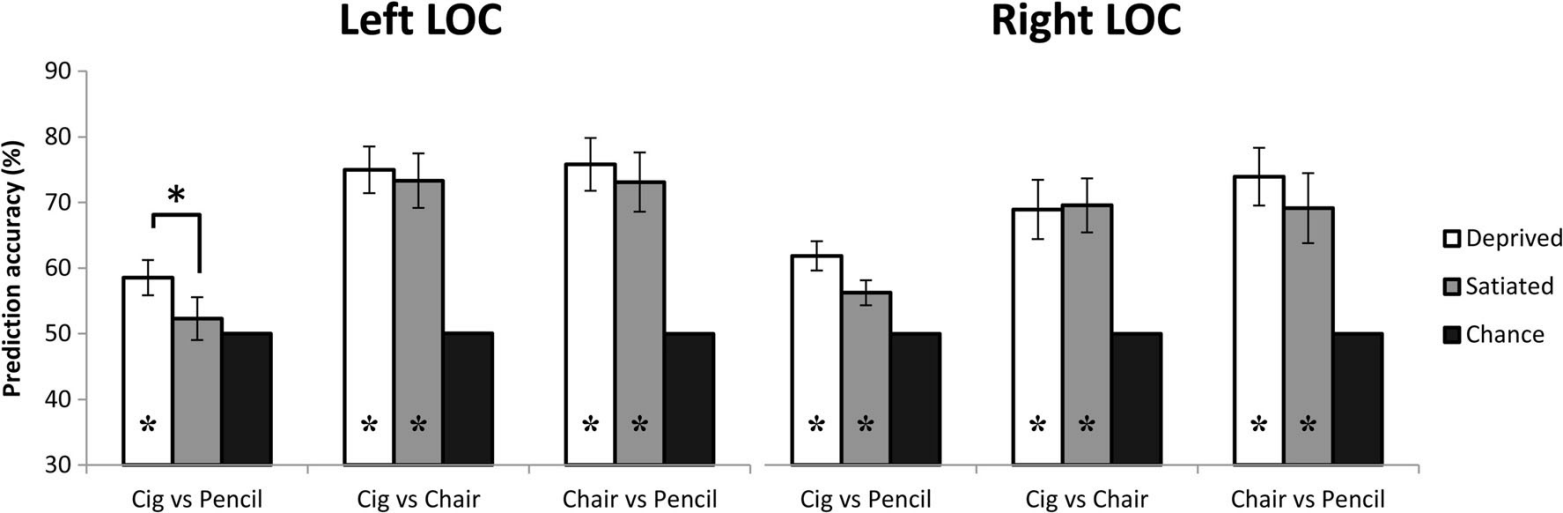
Classification results of all contrasts in left and right LOC separately. In left LOC, decoding accuracies for the cigarette–pencil contrast were significantly higher in the deprived than in the satiated condition (*p* < .01). For all other stimulus contrasts, there was no difference in discriminability between the deprived and satiated conditions, in both left and right LOC. *Asterisks* indicate significant differences from permuted chance levels

In right LOC also, decoding accuracies for the cigarette–pencil contrast were significantly higher than empirically estimated chance levels in both the satiated (mean = 56.3%, mean permutation = 50.0%, *p* = .01 (uncorrected, *p* = .02 FDR-corrected)) and deprived conditions (mean = 61.9%, mean permutation = 50.0%, *p* = .004 (uncorrected, *p* = .006 FDR-corrected)). Moreover, they did not significantly differ between the conditions (accuracies D-S = 5.63, permutations D-S = 0.05, ns.). Similarly, discrimination between cigarettes and chairs was possible above permuted chance levels in satiated (mean = 69.6%, mean permutations = 50.0%, *p* = .004 (uncorrected, *p* = .006 FDR-corrected)) and deprived (mean = 69.0%, mean permutation = 50.0%, *p* = .004 (uncorrected, *p* = .006 FDR-corrected)) participants, and did not differ between conditions (accuracies D-S = −0.63, permutations D-S = −.01, ns.). Furthermore, the classifier was able to discriminate between responses in right LOC to two different neutral objects (pencils and chairs) under conditions of smoking deprivation (mean = 74%, mean permutation = 50.0%, *p* = .004 (uncorrected, *p* = .006 FDR-corrected)) as well as satiety (mean = 69.2%, mean permutation = 50.0%, *p* = .004 (uncorrected, *p* = .006 FDR-corrected)). The classifier’s ability to discriminate between two neutral objects did not differ between the satiated and deprived conditions (accuracies D-S = −.63, permutation D-S = 0.03, ns). There was no significant interaction between the two deprivation states and the three stimulus combinations in right LOC (*p* > .1). Classification results of left and right LOC are depicted in Fig. 4.

Over all stimulus conditions, we did not find the D-S effect to be different across hemispheres. Moreover, when we specifically focussed on the pencil vs cigarette comparison, we again did not find any significant lateralization effects.

## Discussion

This study investigated to what extent nicotine deprivation affects early visual processing of smoking-related objects. More specifically, we examined whether the neural representations of images related to smoking become more apparent when smokers are in a state of abstinence. By means of multi-voxel pattern analysis, we demonstrated (1) that we are able to discriminate between the visually similar categories cigarettes and pencils above chance level by observing the underlying patterns of activity in object-sensitive LOC, and (2) that this discriminability ceases when subjects are satiated. From this, we conclude that the effect of abstinence on behaviour can at least be traced back to a basic level of visual object processing. This indicates that the mechanism by which a smoker’s attention is biased towards smoking cues potentially affects processing in the early visual system, and is not confined to only higher-order motivation and reward-related areas as is generally emphasized in the literature (Volkow et al. 2013; Hester and Luijten 2013; Jasinska et al. 2014). In light of the increased salience of smoking-related cues after nicotine deprivation (Robinson and Berridge 2008), our findings offer a new insight in the possible neural mechanisms that facilitate this behavioural effect.

However, in light of recent discussions about the interpretability of MVPA results, it is important to note that certain inferences about the nature of the data are not trivial to make. For instance, work by Todd and colleagues demonstrates that since MVPA is insensitive to the directionality of an effect, group tests on summary statistics can potentially introduce confounds (Todd et al. 2013). In addition, Davis et al. used simulations to point out that a significant decoding performance does not allow for drawing conclusion about the underlying nature of the representations (Davis et al. 2014). However, our experimental design is set up to address the question whether certain neural responses are affected by the level of nicotine deprivation. Although we cannot draw firm conclusions on the directionality of the effect, we interpret our results in light of what is known about the psychological effect of addictive substances. If the multivariate distance between two object categories (one of which is related to the addiction) increases as an effect of nicotine deprivation, it is not the statistics that drive the interpretation of directionality. Rather, it is the contextual framing of the study that provides us with a potential interpretation of these results.

To further support our interpretation, it would be interesting to compare our results with a non-smoking control group; if non-smokers would not exhibit a similar difference in decoding between smoking-related and neutral objects as we found in smokers, this would confirm our hypothesis that the difference we found was caused by nicotine deprivation. However, if they *would* show a difference in decoding, that would indicate that this difference is inherently present and diminishes as a consequence of acute nicotine consumption.

The fact that we only found an effect of nicotine deprivation on neural processing in the cigarette–pencil contrast is possibly caused by their visually similar appearances. That is, the other smoking vs neutral stimulus contrast consisted of cigarettes and chairs, which have much more distinct visual features. As LOC is specifically sensitive to object shapes (Grill-Spector et al. 2001; Kim et al. 2009), it is plausible that the large difference in shapes between cigarettes and chairs has caused a ceiling effect, due to which differences between the deprived and satiated conditions could not be demonstrated. The large dissimilarity in shape is also very likely to be the reason for the much higher prediction accuracies in the cigarette–chair, as well as the pencil–chair conditions, compared to the cigarette–pencil condition.

A few considerations should be taken into account with regard to the design of our study. First of all, our final sample size of 10 participants is small, even for an fMRI study. Small samples can be problematic for MVPA as they can lead to decoding accuracies that overshoot chance level merely by chance (Combrisson and Jerbi 2015). We therefore chose for a conservative way to quantify our results: Instead of testing our classification results against chance level (i.e. 50%), we have empirically estimated the distribution of prediction accuracies when there is no relationship between the conditions and underlying response patterns. By testing our true prediction accuracy against the accuracies that resulted from this bootstrapping approach, we overcome this problem.

Secondly, our participants exhibited only low (to moderate) levels of nicotine dependence, as indicated by their low FTND scores. Therefore, it is possible that the smoking cues were not as attractive or attention-grabbing for them as they would have been for highly dependent individuals. Hence, for more dependent smokers, patterns of activity related to smoking cues may have been better discriminable from those related to neutral cues. Moreover, highly dependent smokers would have been more affected by the nicotine deprivation condition, possibly increasing discriminability between smoking and neutral cues in the deprived condition even more. In addition, it is striking that our deprivation condition was not successful in inducing craving or other withdrawal symptoms. This could be due to a too short, and overnight, abstinence period, during which participants only missed a few cigarettes. For instance, an abstinence period of 16 h has been shown to reliably induce craving (Jarvik et al. 2000), while our participants were abstinent for only 12 h on average. Although this difference is small, this indicates that longer periods of abstinence (in heavier smokers) may lead to more distinct responses to smoking-related and neutral cues in LOC.

Moreover, differences between participants in metabolism, patterns of cigarette consumption, smoking history and time since last cigarette could have introduced significant variability due to the acute effects of nicotine. Future studies similar to ours, analysing subtle differences in processing between smoking and neutral cues, should control for these factors as much as possible.

Finally, to control as much as possible for other influences, we have kept our images of smoking-related and neutral objects very ‘clean’. They consisted of just one object in a neutral colour presented centrally on a white background. This may have made our smoking images less attractive and interesting to look at than more lifelike images of smoking scenes in which people enjoy smoking cigarettes. Moreover, the fact that our images did not reflect actual smoking scenes may have compromised the ecologic validity of the study.

Nevertheless, we have established that neural response patterns in left LOC differ for smoking-related and neutral pictures when participants were deprived of nicotine. This indicates that the well-known attention bias that has often been reported in smokers likely affects basic visual object processing. This finding may provide a new target for smoking cessation interventions; for instance, non-invasive brain stimulation techniques may be used to manipulate brain activity in LOC. Moreover, it shows that treatment interventions should not just aim to eliminate craving elicited by smoking cues, but should specifically focus on extinction of automatic responses to these cues.
